# A gonadal gap junction INX-14/Notch GLP-1 signaling axis suppresses gut defense through an intestinal lysosome pathway

**DOI:** 10.3389/fimmu.2023.1249436

**Published:** 2023-10-19

**Authors:** Xiumei Zhang, Yirong Wang, Zixin Cai, Zhiqing Wan, Yilixiati Aihemaiti, Haijun Tu

**Affiliations:** State Key Laboratory of Chemo/Biosensing and Chemometrics, College of Biology, Hunan University, Changsha, Hunan, China

**Keywords:** reproductive tract, gap junction, gut defense, notch signaling, lysosome pathway

## Abstract

Gap junctions mediate intercellular communications across cellular networks in the nervous and immune systems. Yet their roles in intestinal innate immunity are poorly understood. Here, we show that the gap junction/innexin subunit *inx-14* acts in the *C. elegans* gonad to attenuate intestinal defenses to *Pseudomonas aeruginosa* PA14 infection through the PMK-1/p38 pathway. RNA-Seq analyses revealed that germline-specific *inx-14* RNAi downregulated Notch/GLP-1 signaling, while lysosome and PMK-1/p38 pathways were upregulated. Consistently, disruption of *inx-14* or *glp-1* in the germline enhanced resistance to PA14 infection and upregulated lysosome and PMK-1/p38 activity. We show that lysosome signaling functions downstream of the INX-14/GLP-1 signaling axis and upstream of PMK-1/p38 pathway to facilitate intestinal defense. Our findings expand the understanding of the links between the reproductive system and intestinal defense, which may be evolutionarily conserved in higher organism.

## Introduction

The mammalian reproductive system not only produces offspring for the maintenance of the species, but also functions innate and adaptive immune response to internal and external stimuli for homeostasis. Interactions between the cells that make up the reproductive immune system and the local microenvironment play crucial roles in the immune responses of the reproductive tract (1). Adaptive immunity generally requires lymphocyte subsets, including B cells, CD4^+^ and CD8^+^ T cells, although there are sex-dependent differences in mammalian reproductive tracts (2). Dimorphic innate immune responses are regulated by cytokines and chemokines produced by local innate immune cells (including natural killer (NK) cells, neutrophils, macrophages), innate lymphoid cells (ILCs) in the reproductive tract, and sex steroid hormones secreted by reproductive tissues (2). Previous studies revealed that intestinal immunity and gut defense are not only controlled by immune cells including macrophages, mast cells and innate lymphoid cells (3), but also regulated by other types of cells such as neurons, muscle cells (3–5). Yet, the role of the reproductive tract in the gut immunity and intestinal defenses remains poorly understood.

The nematode *Caenorhabditis elegans* provides a powerful genetic model to dissect the mechanisms of host-pathogen interactions, innate immune response and intestine defense (6). Although they lack classical adaptive immunity, worms sense pathogen-derived cues (7, 8), exhibit pathogen avoidance behaviors (9, 10) and trigger powerful innate immune responses to defend against pathogen infections (11–13). To date, several evolutionarily conserved signaling pathways have been implicated in *C. elegans’* response to diverse pathogens, including PMK-1/p38 mitogen-activated protein kinase (MAPK) (13), insulin/IGF-1 signaling (IIS) (14), and DBL-1/transforming growth factor (TGF-β) pathways (11). Activation of these pathways stimulates the expression of immune effectors, including lysozymes, lectins, and antimicrobial peptides in intestinal epithelial cells, which function to eliminate invading pathogens (15).

Gap junctions, also known as electrical synapses, are intercellular channels that are widely expressed in vertebrate and invertebrate tissues (16–18). Gap junctions are comprised of two hemichannels embedded in two adjacent cell membranes that dock to form a complete channel and provide a pathway for the exchange of small signaling molecules and ions in a controlled manner. As in higher organisms, *C. elegans* gap junctions allow direct cell-to-cell communication based on membrane voltage, nutrient and second messengers exchange (including Ca^2+^, IP3 and the cyclic nucleotides cAMP and cGMP), and metabolic signal transmission across cell membranes (19–24).

Previous studies estimated that *C. elegans* forms ∼600 gap junctions between its 302 neurons and that gap junction components are involved in diverse biological processes such as establishment of neuronal asymmetry, mechanosensation, aggregation, and aversive olfactory behaviors (25–28). For instance, the innexin gap junction protein NSY-5 coordinates left-right asymmetry of AWC olfactory neurons to establish stochastic, asymmetric gene expression patterns (25). *inx-4* encodes an innexin expressed in the RIM neuron, and RIM-expressed NMR-1 mediates olfactory training-dependent downregulation of INX-4 in PA14-induced neuronal responses (26). Although an increasing number of studies reveal the importance of gap junctions in the nervous system, the role of gap junctions in the intestine defense is poorly understood.

Here, we systematically investigated the susceptibility of gap junction loss-of-function mutants to *Pseudomonas aeruginosa* PA14 infection and identified that the innexin gene *inx-14*, encoding a heterotypic and heteromeric gap junction subunit, acts in the gonad to suppress intestinal defense through a PMK-1/p38 pathway. Transcriptome profiling revealed that GLP-1/Notch signaling was downregulated when *inx-14* was knocked-down in the germline by RNAi, while lysosome pathways, and PMK-1/p38 MAPK signaling were upregulated. We further demonstrate that gonadal INX-14-mediated GLP-1/Notch signaling suppresses the lysosome pathway to regulate gut defense in a PMK-1/p38-dependent manner. Our findings reveal the important roles of the reproductive tract-intestinal epithelia unit in the response to *Pseudomonas aeruginosa* PA14 infection, a process that may be evolutionarily conserved in higher organisms.

## Results

### Loss of the heterotypic and heteromeric gap junction subunit *inx-14* causes enhanced resistance to pathogenic *P. aeruginosa* PA14 infection

**Figure 1:**
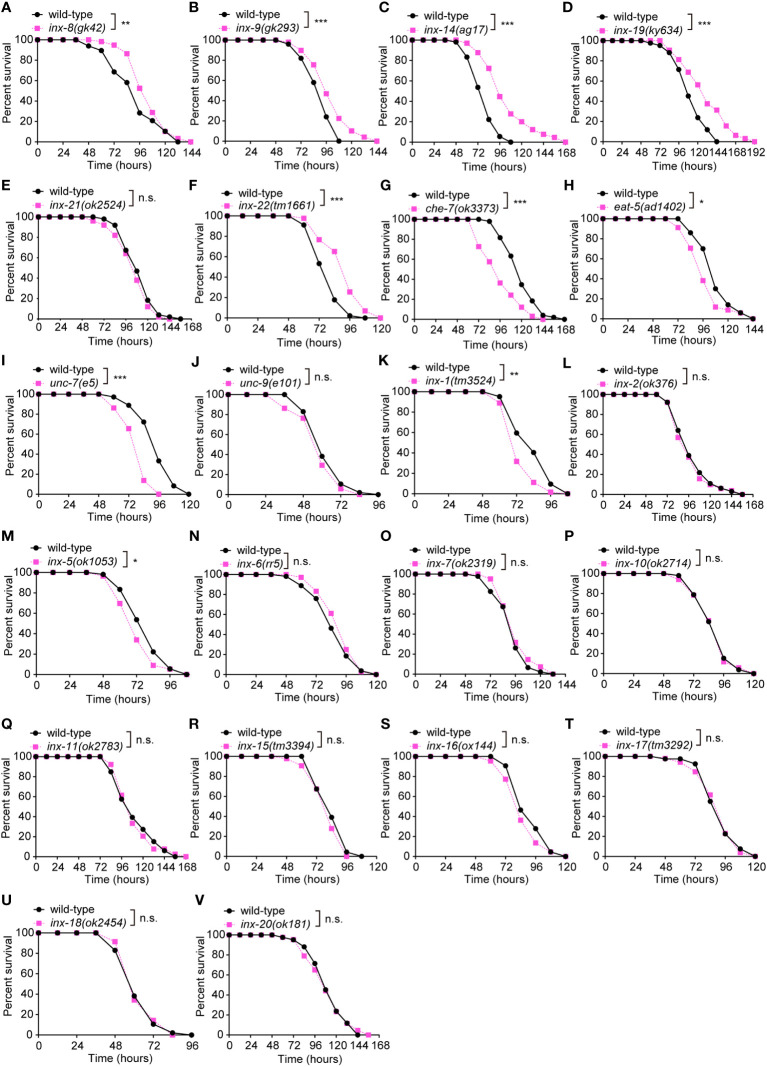
Loss of the gonadal heterotypic heteromeric gap junction subunit gene *inx-14* causes enhanced resistance to *P. aeruginosa* PA14 infection. (A – F) PA14 survival assays of wild-type and gap junction subunit gene mutants *inx-8(gk42)* **(A)**, *inx-9(gk293)* **(B)**, *inx-14(ag17)* **(C)**, *inx-19(ky634)* **(D)**, *inx-21(ok2514)* **(E)**, and *inx-22(tm1661)* **(F)**. **(G–V)** Survival assays of wild-type and gap junction subunit gene mutants *che-7(ok3373)* **(G)**, *eat-5(ad1402)* **(H)**, *unc-7(e5)* **(I)**, *unc-9(e101)* **(J)**, *inx-1(tm3524)* **(K)**, *inx-2(ok375)* **(L)**, *inx-5(ok1053)* **(M)**, *inx-6(rr5)* **(N)**, *inx-7(ok2319)* **(O)**, *inx-10(ok2714)* **(P)**, *inx-11(ok2783)* **(Q)**, *inx-15(tm3394)* **(R)**, *inx-16(ox144)* **(S)**, *inx-17(tm3292)* **(T)**, *inx-18(ok2454)* **(U)**, and *inx-20(ok181)* **(V)**. All experiments were repeated at least three times. Statistical significance was determined by log-rank test. *P < 0.05; **P < 0.01; ***P < 0.001; n.s., not significant. The exact P values of statistics for all survival assays are listed in **Supplementary Table S1**.

The *C. elegans* gap junction gene family is composed of 25 innexin subunits. While these proteins share no primary sequence homology with vertebrate connexins, they exhibit clear structural and functional similarities (19, 29, 30). Each gap junction is formed by oligomerization of six subunits on each cell membrane. Channel subunits can come together in homomeric or heteromeric combinations and form homotypic or heterotypic assemblies (31). The 25 gap junction subunits form channels that are defined by the combinatorial expression of specific innexin proteins in *C. elegans* (31–33). To investigate whether gap junctions modulate innate immunity, we challenged loss-of-function mutants of innexin genes with the well-established *Pseudomonas aeruginosa* PA14 survival assays (**Figures 1A–V**). These experiments revealed different classes of innexin genes involved in pathogen resistance. First, loss-function mutants for *che-7*, *eat-5*, *inx-1*, *inx-5, or unc-7* exhibited enhanced susceptibility to PA14 infection (**Figures 1G–I, K, M**). In contrast, we observed increased resistance for loss-of-function mutants of *inx-8*, *inx-9*, *inx-14*, *inx-19* or *inx-22* (**Figures 1A–D, F**). We however failed to identify any obvious effects for 12 additional innexins (*unc-9*, *inx-2*, *inx-6, inx-7, inx-10, inx-11, inx-15, inx-16, inx-17, inx-18, inx-20* and *inx-21*) (**Figures 1J, L, N–V, E**). Finally, we did not assay three essential innexin genes (*inx-3*, *inx-12* and *inx-13*) as no viable mutants are available (34–36). Taken together, these results clearly implicate multiple innexins in innate intestinal defense in *C. elegans*.

Previous studies have demonstrated that INX-8, INX-9, INX-14, INX-21 and INX-22 form two classes of gap junction channels in distinct combinations. Germline proliferation defect of *inx-14* loss-of-function mutants can be phenocopied by *inx-8;inx-9* or *inx-22;inx-21* double mutants (16), suggesting that *inx-14* is essential for these two classes of channels. This prompted us to focus our attention on the role of *inx-14* for intestinal defense upon *Pseudomonas aeruginosa* PA14 infection.

### INX-14 functions in the gonad to suppress intestinal defense against PA14 infection

**Figure 2:**
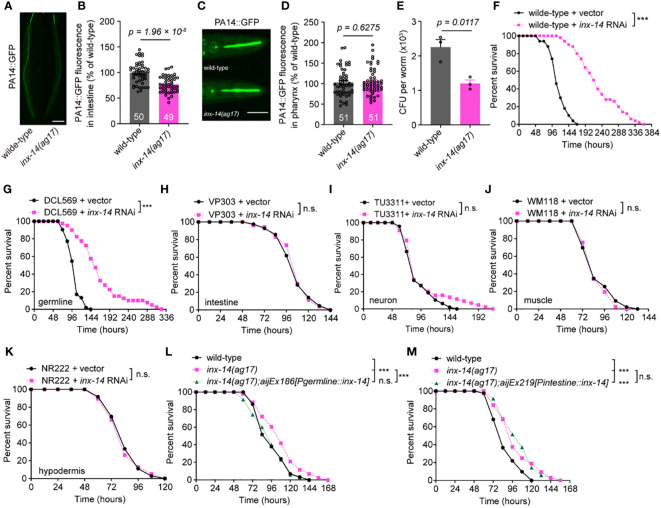
INX-14 acts in the germline of *C elegans* to suppress resistance to PA14. **(A–D)** Representative images **(A, C)** and fluorescence intensity **(B, D)** of GFP-expressing PA14 bacteria accumulated in the intestine **(A, B)** or pharynx **(C, D)** of wild-type and *inx-14(ag17)* mutants after exposed to *P. aeruginosa* for 24 (h) **(E)** Quantification of the colony-forming units (CFU) of wild-type and *inx-14(ag17)* worms exposed to PA14. Data are presented as mean ± SEM. **(F)** Survival of wild-type worms fed on vector control or *inx-14* RNAi bacteria prior to PA14 exposure. **(G–K)** PA14 survival assays following tissue-specific RNAi in the germline (DCL569) **(G)**, intestine (VP303) **(H)**, neurons (TU3311) **(I)**, muscle (WM118) **(J)**, hypodermis (NR222) **(K)** of animals fed on vector control or *inx-14* RNAi bacteria prior to PA14 exposure. **(L, M)** Survival assay of wild-type, *inx-14(ag17)*, and transgenic lines expressing INX-14 in the germline **(L)** and in the intestine **(M)**. All experiments were repeated at least three times. The number of animals analyzed is indicated in **(B)** and **(D)**. Scale bar, 10 μm. Statistical significance was determined by log-rank test for survival assays, or unpaired Student’s t-test for **(B)**, **(D)**, or non-parametric test for **(E)**. ***P < 0.001; n.s., not significant. The exact P values of statistics for all survival assays are listed in **Supplementary Table S1**.

Since pathogen avoidance is an important behavior for reducing stress caused by microbial infection and for survival to pathogen invasion (37), we compared the avoidance of wild-type and *inx-14(ag17)* mutants to pathogens during PA14 infection. We found that the percentages of occupancy on a PA14 lawn were indistinguishable between *inx-14(ag17)* mutants and wild-type animals at 2, 4, 6, 8, and 10 hours after exposure to a lawn of PA14 bacteria (**Figure S1A**). *inx-14(ag17)* mutants also displayed normal enteric muscle contractions (**Figure S1B**), pharyngeal pumping (**Figure S1C**), body bends (**Figure S1D**), and distribution of green fluorescent beads in the intestine when fed with a mixture of green fluorescent beads and PA14 (**Figures S2A**, **S2B**). However, *inx-14* mutants exhibited significantly longer survival (**Figure S2C**) compared to wild-type animals. In some *C. elegans* mutants, resistance to PA14 infection is linked to reduced accumulation of pathogenic bacteria in the intestine (38, 39). We therefore examined PA14 gut content of *inx-14(ag17)* after infection. Our results showed that GFP-labeled PA14 bacteria (PA14::GFP) accumulated less in the intestine of *inx-14(ag17)* mutants compared to wild-type. However, accumulation was not different in the pharynx (**Figures 2A–D**). Consistently, the number of PA14 bacteria in the body of *inx-14(ag17)* mutant as assayed by colony forming units was dramatically decreased compared to wild-type (**Figure 2E**). These results suggest that the loss of INX-14 enhances the worm’s capacity to eliminate pathogenic bacteria.

The innate immune response induced by bacterial infection occurs primarily in the gut (6) or epidermal tissue (11) of *C. elegans*. Remarkably, *inx-14* is mainly expressed in the germline, neurons and muscle cells (34, 40). To determine in which tissue *inx-14* acts to reduce intestinal defense, we treated animals with tissue-specific *inx-14* RNAi prior to PA14 infection. First, we found that wild-type animals fed with *inx-14* RNAi bacteria displayed robust resistance to PA14 infection (**Figure 2F**). Next, we observed that tissue-specific *inx-14* RNAi in the germline (DCL569) was sufficient to phenocopy this effect (**Figure 2G**), while RNAi in the intestine (VP303) (**Figure 2H**), neurons (TU3311) (**Figure 2I**), muscle (WM118) (**Figure 2J**) or hypodermis (NR222) (**Figure 2K**) did not. Furthermore, the enhanced resistance of *inx-14(ag17)* mutants to PA14 infection was fully rescued by *inx-14* transgene expression in the germline under the control of germline-specific *pgl-1* promoter (**Figure 2L**), but not in the intestine under the control of intestine-specific *ges-1* promoter (**Figure 2M**). Finally, we also observed enhanced resistance to PA14 infection in *inx-14(ag17)* males (**Figure S2D**), or larvae at different life stages (L1, L2, or L3) (**Figures S2E–S2G**). Taken together, these results supported the notion that *inx-14* expressed in the germline may suppress intestinal defense response against pathogen infection.

### Gonad gap junction-dependent intestinal defense requires PMK-1/p38, but not FOXO/DAF-16 or TGF-β/DBL-1 immune pathways

**Figure 3:**
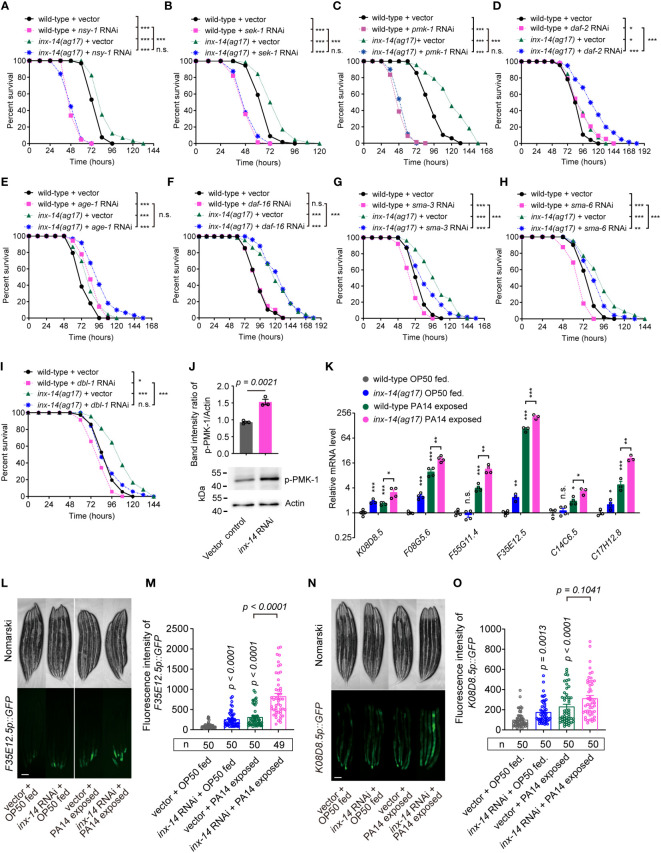
Resistance of inx-14 loss-of-function mutants to PA14 infection requires the PMK-1/p38 pathway, but not FOXO/DAF-16 or TGF-β/DBL-1 immune pathways. **(A–C)** PA14 survival assays of wild-type and *inx-14(ag17)* mutants fed with vector control, or RNAi against PMK-1/p38 pathway genes *nsy-1* **(A)**, *sek-1* **(B)**, *pmk-1* **(C)**. **(D–F)** Survival assays of wild-type and *inx-14(ag17)* mutants fed on vector control or RNAi against IIS/DAF-16 pathway component genes *daf-2* **(D)**, *age-1* **(E)**, *daf-16* **(F)**. **(G–I)** Survival assays of wild-type and *inx-14(ag17)* mutants fed on vector control or RNAi targeting DBL-1/TGF-β pathway components *sma-3* **(G)**, *sma-6* **(H)**, *dbl-1* **(I)**. **(J)** Representative western blots and quantitative analysis of the phosphorylation level of PMK-1/p38 in wild-type animals fed with vector control or *inx-14* RNAi prior to PA14 infection for 24 (h) **(K)** RT-qPCR analyses of the expression levels of six PMK-1/p38-dependent genes in wild- type and *inx-14(ag17)* mutant animals fed on OP50 or exposed to PA14. The exact P values of statistics for comparison of qRT-PCR between groups are listed in **Supplemental Table S2**. **(L–O)** Representative images **(L, N)** and quantification **(M, O)** of PMK-1/p38 pathway-dependent gene reporters F35E12.5p::GFP **(L, M)** or K08D8.5p::GFP **(N, O)** fed on vector control or *inx-14* RNAi bacteria prior to OP50 feeding or PA14 exposure. All experiments were repeated at least three times. Column data with plots are presented as mean ± SEM. The number of animals analyzed is indicated. Statistical significance was determined by log-rank test for survival assays, or non-parametric test for **(J)**, or Kruskal–Wallis with Dunn’s multiple comparison test for **(K, M, O)**. *P < 0.05; **P < 0.01; ***P < 0.001; n.s., not significant. Scale bar, 100μm in **(L, N)**.

Previous studies have revealed that several evolutionarily conserved signaling pathways are required for *C. elegans* defense response to diverse pathogens, including the PMK-1/p38 mitogen-activated protein kinase (MAPK) (13), insulin/IGF-1 signaling (IIS) (14), and DBL-1/transforming growth factor (TGF-β) pathways (11). Activation of these pathways triggers the expression of immune effectors such as lysozymes, lectins, and antimicrobial peptides in intestinal epithelial cells, which recognize and eliminate invading pathogens (15). To examine which signaling pathways are required for INX-14-mediated intestinal defense, we knocked down the expression of essential components of these pathways using RNAi in an *inx-14(ag17)* mutant background and compared their survival on PA14 bacteria. We observed that the resistance of inx-14(ag17) mutant to PA14 was almost completely suppressed by RNAi knockdown of the PMK-1/p38 pathway genes *nsy-1*, *sek-1*, and *pmk-1* (**Figures 3A–C**). This genetic interaction suggested that *inx-14* may act upstream of the PMK-1/p38 pathway.

It has previously been reported that mutants for *daf-2* (insulin/IGF receptor) and *age-1* (catalytic subunit of phosphatidylinositol 3-OH kinase) exhibit enhanced resistance to PA14 infection (41), similarly to *inx-14* mutants described here. When, we performed *daf-2* or *age-1* RNAi knockdown in *inx-14(ag17)* mutants, we observed a significantly stronger resistance to PA14 than in either single mutant (**Figures 3D, E**), while *daf-16* RNAi in wild-type or *inx-14(ag17)* mutant animal did not alter the sensitivity of these strains (**Figure 3F**).

In contrast, RNAi-mediated knock-down of the DBL-1/TGF-β pathway genes *sma-3*, *sma-6*, or *dbl-1*, enhanced sensitivity of wild-type worms to PA14 infection (**Figures 3G–I**). This enhancement of sensitivity to PA14 was conserved in an *inx-14(ag17)* genetic background (**Figures 3G–I**).

We next examined the phosphorylation level of p38/PMK-1 and found that it was significantly increased by *inx-14* RNAi (**Figure 3J**). Consistently, RT-qPCR analyses revealed that six p38/PMK-1-dependent genes, including *K08D8.5* (43), *F08G5.6* (11), *F55G11.4* (42), *F35E12.5* (44), *C14C6.5* (42) and *C17H12.8* (45) were significantly up-regulated upon PA14 infection in wild-type and *inx-14(ag17)* mutants (**Figure 3K**). The fluorescence intensities of the p38/PMK-1-dependent gene reporters *F35E12.5p::GFP* and *K08D8.5p::GFP* were also dramatically increased by *inx-14* RNAi (**Figures 3L–O**). Together these results suggest that INX-14-mediated intestinal defense of *C. elegans* is dependent on the PMK-1/p38, but not IIS/DAF-16, or DBL-1/TGF-β signaling pathways, and that the PMK-1/p38 pathway acts downstream of INX-14 to regulate intestinal defense.

### GLP-1/Notch signaling acts downstream of INX-14 in the germline to suppress intestine defense

To elucidate the mechanism of INX-14-regulated intestinal defense, we performed a transcriptomic analysis in the germline-specific RNAi strain DCL569 (46) fed with control or *inx-14* RNAi, prior to OP50 feeding or PA14 infection. Differential gene expression analyses of RNA-Seq data revealed that *inx-14* RNAi treatment led to downregulation of 4757 genes upon PA14 exposure (**Figure S3A** and datasets S1), and down-regulation of 56 genes when fed with OP50 (**Figure S3A**). The KEGG pathway enrichment analysis of the 4757 down-regulated genes revealed that the Notch signaling pathway (marked with a red asterisk) (**Figure S3B**) was significantly downregulated.

**Figure 4:**
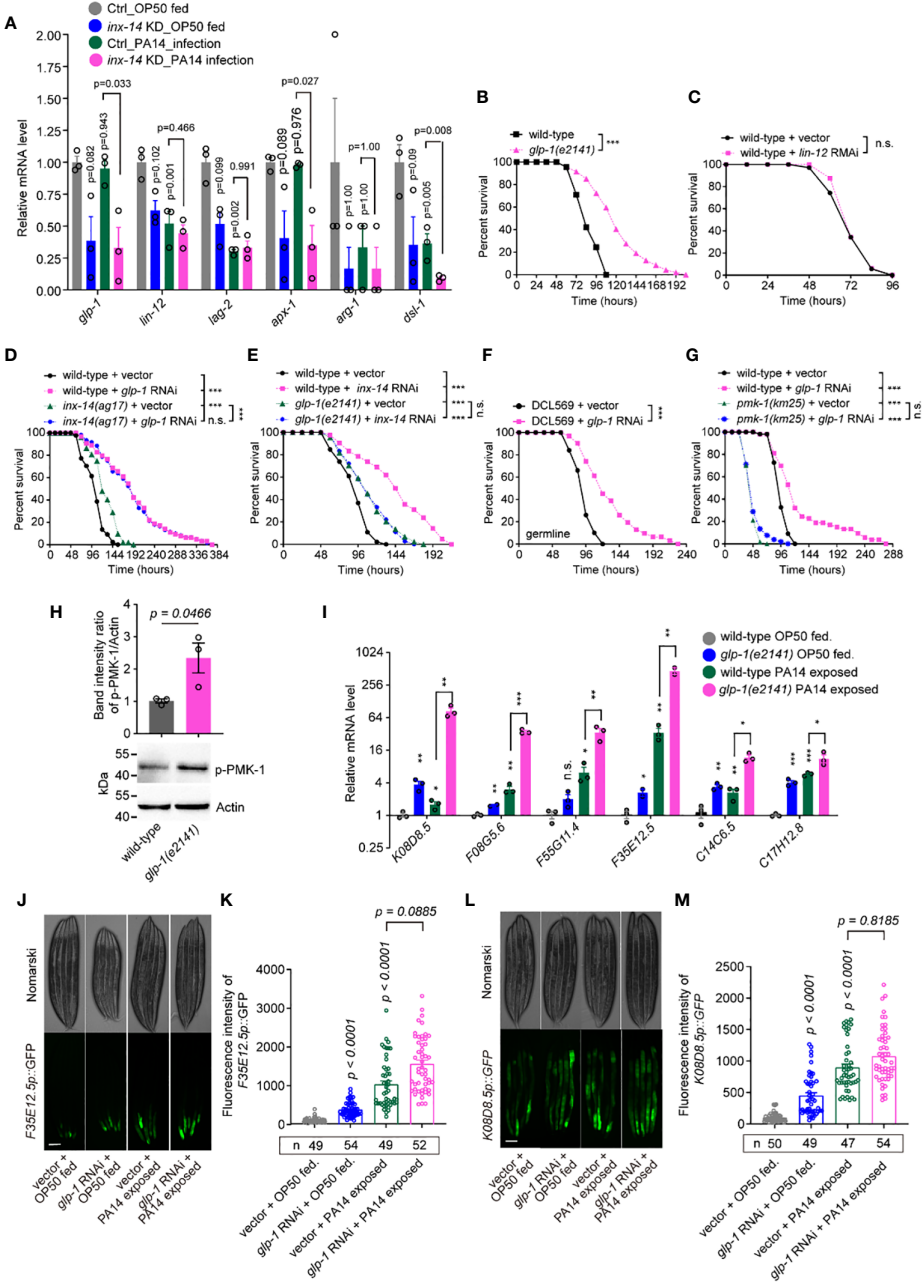
GLP-1/Notch signaling acts downstream of INX-14 in the germline to suppress the intestinal defense. **(A)** Normalized expression levels of Notch signaling pathway components *glp-1*, *lin-12*, *lag-2*, *apx-1*, *arg-1* and *dsl-1* in the transcriptome profile of germline-specific RNAi strain DCL569. Ctrl, L4440 vector control; *inx-14* KD indicates *inx-14* knockdown by germline-specific RNAi. **(B, C)** Survival assay of wild-type and glp-1(e2141) mutant **(B)**, or of animals fed on vector control or *lin-12* RNAi bacteria **(C)**. **(D)** Survival assay of wild-type and *inx-14(ag17)* mutant animals fed on vector control or *glp-1* RNAi bacteria. **(E)** Survival assays of wild-type and *glp-1(e2141)* mutants fed on vector control or *inx-14* RNAi bacteria, prior to PA14 exposure. **(F)** Survival assays of the germline-tissue-specific RNAi strain DCL569 fed on vector control or *inx-14* RNAi bacteria prior to PA14 exposure. **(G)** Survival assays of wild-type and *pmk-1(km25)* mutant animals fed on vector control or *glp-1* RNAi bacteria, prior to PA14 exposure. **(H)** Representative western blot images and quantitative analysis of the phosphorylation level of PMK-1/p38 in wild-type and *glp-1(e2141)* mutant animals upon PA14 exposure. **(I)** RT-qPCR analysis of the expression levels of six PMK-1/p38-dependent genes in wild-type and *glp-1(e2141)* mutants fed on OP50 or exposed to PA14. The exact P values of statistics for comparison of qRT-PCR between groups are listed in **Supplementary Table S2**. **(J–M)** Representative images **(J, L)** and quantification **(K, M)** of PMK-1/p38 pathway-dependent gene reporters F35E12.5p::GFP **(J, K)** and K08D8.5p::GFP **(L, M)**, fed on vector control or *inx-14* RNAi bacteria, prior to OP50 feeding or exposure to PA14. All experiments were repeated at least three times. The number of animals analyzed is indicated in **(K, M)**. Column data with plots are presented as mean ± SEM. Statistical significance was determined by log-rank test for survival assay, or non-parametric test for **(H)**, or Kruskal–Wallis with Dunn’s multiple comparison test for **(I)**, **(K)**, and **(M)**. *P < 0.05; **P < 0.01; ***P < 0.001; n.s., not significant. Scale bar, 100 μm in **(J, L)**.

Based on these RNA-Seq data, we observed that the mRNA expression levels of *glp-1* and *lin-12* (two Notch receptor orthologs), *apx-1* and *lag-2* (two Notch ligands), and *arg-1* and *dsl-1* (encoded Notch binding activity) (47) were down-regulated by *inx-14* RNAi (**Figure 4A**). Previous studies found that the GLP-1/Notch pathway regulates *C. elegans* germline development (48) and loss of *glp-1* caused enhanced resistance to PA14 infection (49). Consistently, we found that a *glp-1(e2141)* mutant, but not *lin-12* RNAi, showed dramatically enhanced resistance to PA14 infection (**Figures 4B, C**). Surprisingly, RNAi knock-down of *lag-2* and *apx-1*, either individually or in combination, did not obviously affect the susceptibility of wild-type animals to PA14 infection (**Figures S3C–S3E**), suggesting that these Notch ligands are dispensable for INX-14-mediated gut defense.

The transforming growth factor β (TGF-β) family is a major evolutionarily-conserved signal transduction pathway that plays crucial roles in development and homeostasis (48, 50, 51). The canonical TGF-β signal transduction pathway is comprised of two transmembrane ser/thr kinase receptors, namely type I TGF-β receptors encoded by *daf-1* or *sma-6*, and a type II receptor encoded by *daf-4*. DAF-7 and DBL-1 are ligands of type I and type II TGF-β receptors, respectively, which are referred to as the Dauer pathway and the Sma/Mab pathway (52). Our results showed that INX-14-mediated intestinal defense is not dependent of the DBL-1/TGF-β signaling pathway (**Figures 3G–I**). Previous studies have found that DAF-7/TGF-β signaling acts in distal tip cells (DTC) to directly regulate the proliferation/differentiation decision in the germline, in parallel with the GLP-1/Notch pathway (48). To address if DAF-7/TGF-β also acts in INX-14-mediated intestinal defense, we examined the survival rate of animals with decreased DAF-7/TGF-β signaling. We found that RNAi knock down of *daf-1*, *daf-3*, *daf-4*, *daf-5* or *daf-7* in wild-type and *inx-14(ag17)* mutants did not significantly alter their susceptibility to PA14 infection (**Figures S3F–S3J**). Together these results suggested that GLP-1/Notch rather than DAF-7/TGF-β signaling is involved in the suppression of the intestinal immunity in *C. elegans*.

To test if and how *glp-1* genetically interacts with *inx-14*, we examined and compared the survival rate of wild-type and *inx-14(ag17)* mutants with vector control or *glp-1* RNAi, upon PA14 infection. We observed that the resistant effect of *inx-14* or *glp-1* RNAi in wild-type animals to PA14 infection was stronger than that of any corresponding *inx-14(ag17)* or *glp-1(e2141)* mutants (**Figures 4D, E**), which maybe result from partial loss function of INX-14 in *inx-14(ag17)* or of GLP-1 in *glp-1(e2141)* mutants for both *ag17* and *e2141* alleles are substitution mutations (53, 54) although we could not rule out the possibility of RNAi off-target effect. We then observed that the survival of *glp-1* RNAi in *inx-14(ag17)* mutants exhibited no significant difference to that of *glp-1* RNAi in wild-type animals (**Figure 4D**). Alternatively, we observed that the survival rate of *inx-14* RNAi in *glp-1(e2141)* mutants blocked the effect of significantly different from that of *inx-14* RNAi in wild-type animals, indicating that loss function of *glp-1* completely blocked the more enhanced resistance of *inx-14* RNAi in wild-type than that of *glp-1(e2141)* mutants (**Figure 4E**). Taken together, these data suggest that *inx-14* and *glp-1* function in the same genetic pathway, and *glp-1* may genetically act downstream of *inx-14* to suppress intestinal defense.

To address in which tissue *glp-1* functions, we examined the susceptibility of tissue-specific *glp-1* RNAi and found that germline-specific RNAi (DCL569) (46) (**Figure 4F**), but not neuron- (TU3311) (55) (**Figure S3K**), muscle- (WM118) (56) (**Figure S3L**), intestine- (VP303) (57) (**Figure S3M**), or hypodermis-specific RNAi (NR222) (58) (**Figure S3N**) caused enhanced resistance to PA14 infection. Collectively, these data suggest that GLP-1/Notch acts in the germline and downstream of INX-14/GAP junction to suppress the intestinal defense of *C. elegans*.

Next, to test a possible link between GLP-1 signaling and the PMK-1/p38 pathway, we examined the effect of loss-of-function mutations for *sek-1*, *nsy-1*, and *pmk-1* on the PA14 susceptibility of wild-type animals fed with *glp-1* RNAi. We found that these mutations completely suppressed the enhanced susceptibility caused by *glp-1* RNAi (**Figure 4G**, **Figures S3O, S3P**).

We also detected an increased phosphorylation level of PMK-1/p38 in *glp-1(e2141)* mutants (**Figure 4H**). Furthermore, as we observed above of *inx-14* (**Figure 3K**), RT-PCR analysis revealed that six PMK-1/p38-dependent genes *K08D8.5* (43), *F08G5.6* (11), *F55G11.4* (42), *F35E12.5* (44), *C14C6.5* (42) and *C17H12.8* (45), were significantly up-regulated upon PA14 infection in wild-type and in *glp-1(e2141)* mutants (**Figure 4I**). Consistently, the fluorescence intensities of the PMK-1/p38-dependent gene reporters *F3512.5p::GFP* and *K08D8.5p::GFP* were dramatically increased either upon PA14 infection or *glp-1* RNAi treatment (**Figures 4J–M**). Taken together, we propose that GLP-1/Notch signaling acts downstream of the INX-14/Gap junction to represses intestinal defense by inhibiting PMK-1-dependent signaling.

### Gonadal INX-14 and GLP-1 repress an intestinal lysosome pathway

**Figure 5:**
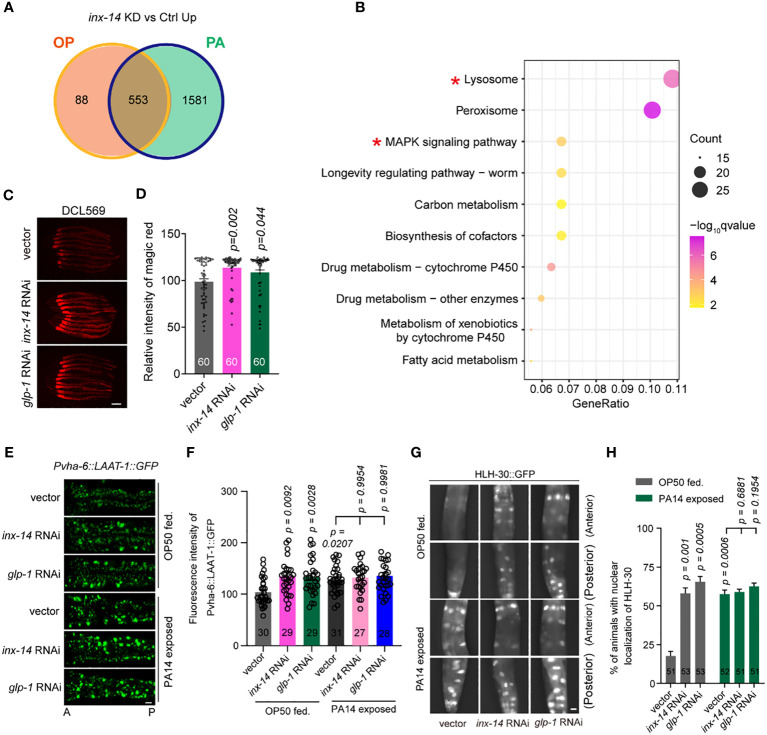
Knock-down of *inx-14* and *glp-1* upregulates a lysosome pathway. **(A)** Venn diagram of genes up-regulated upon germline-specific *inx-14* knock-down in OP50 or PA14 exposed animals. **(B)** KEGG pathway enrichment analyses of the up-regulated genes upon PA14 infection and *inx-14* RNAi knock-down. Two red asterisks mark the MAPK signaling and lysosome pathways. KEGG terms were sorted according to numbers of enriched differentially-expressed genes. **(C, D)** Representative images **(C)** and quantitative analysis **(D)** of Magic Red Cathepsin assay in the germline-specific RNAi strain DCL569. **(E–H)** Representative images **(E, G)** and quantitative analysis **(F, H)** of transgenic worms expressing LAAT-1::GFP in the intestine **(E, F)**, or HLH-30::GFP **(G, H)** fed on vector control, *inx-14*, or *glp-1* RNAi bacteria, prior to OP50 feeding or PA14 infection for 24 (h) Statistical significance was determined by Kruskal–Wallis with Dunn’s multiple comparison test and the exact P value is indicated for **(D, F, H)**. Scale bar, 100 μm in **(C, E, G)**. Two red asterisks mark the enriched signaling pathways of lysosome and MAPK signaling pathways, respectively.

To gain insight into the cellular mechanisms underlying INX-14 and GLP-1-mediated intestinal defense, we performed KEGG pathway enrichment analyses of the genes that were up-regulated upon PA14 infection in *inx-14* RNAi treated animals compared to wild-type (**Figures 5A**, **Datasets S2**). This analysis revealed significant enrichment of MAPK signaling and lysosome pathways (**Figures 5B**, **Datasets S3**). These results were consistent with our data showing that INX-14/GLP-1-mediated intestinal defense is dependent on PMK-1/p38 MAPK signaling (**Figure 3**).

Next, we focused on the role of the lysosome pathway in intestinal defense. We first examined whether lysosomal function was affected by INX-14/GLP-1 signaling using the Magic Red Cathepsin assay (59). We found that germline-specific knockdown of *inx-14* or *glp-1* by RNAi increased Magic Red staining fluorescence intensity (**Figures 5C, D**). Second, we observed that the expression of LAAT-1, a lysosomal lysine/arginine transporter specifically expressed in the gut using the *vha-6* promoter (60, 61), was increased upon PA14 infection, or by RNAi knock-down of *inx-14* or *glp-1* (**Figures 5E, F**).

Finally, previous studies have revealed that the transcription factor HLH-30, a homolog of mammalian TFEB, is a master regulator of lysosomal biogenesis (62–64). Nuclear translocation of HLH-30 induced by extra- and intracellular stress conditions is a critical step that activates transcription of genes involved in lysosome biogenesis (59, 64, 65). Thus, we examined the nuclear localization of GFP-tagged HLH-30 (HLH-30::GFP) and found that the portion of nuclear HLH-30::GFP localization dramatically increased upon PA14 infection, or upon *inx-14* or *glp-1* RNAi treatment (**Figures 5G, H**). Collectively, these data suggest that a INX-14/GLP-1 signaling axis in the germline may control intestinal lysosome activity in a cell non-autonomous manner.

### Intestinal lysosome facilitates gut defense through a PMK-1/p38 pathway

**Figure 6:**
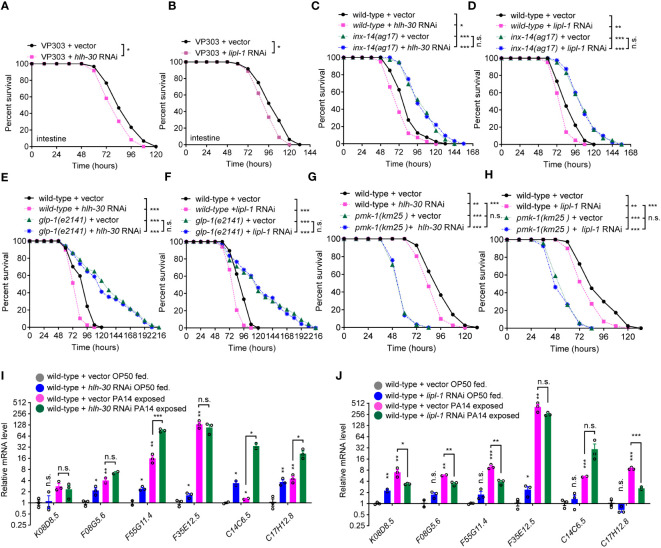
A lysosome pathway is involved in intestinal defense via PMK-1/p38. **(A, B)** PA14 survival assays of the intestine-specific RNAi strain VP303 fed on vector control, *hlh-30* **(A)**, *lipl-1* **(B)** RNAi bacteria, prior PA14 exposure. **(C, D)** Survival assays of wild-type and *inx-14(ag17)* mutant animals fed on vector control, *hlh-30* **(C)**, or *lipl-1* **(D)** RNAi bacteria, prior to PA14 exposure. **(E, F)** Survival assays of wild-type and *glp-1(e2141)* mutants fed on vector control, *hlh-30* **(E)**, or *lipl-1* **(F)** RNAi bacteria, prior to PA14 exposure. **(G, H)** Survival assays of wild-type and *pmk-1(km25)* mutants fed on vector control, *hlh-30* **(G)**, or *lipl-1* **(H)** RNAi bacteria, prior to PA14 exposure. **(I, J)** qRT-PCR analyses of the expression levels of six PMK-1/p38-dependent genes in wild-type animals fed on vector control, *hlh-30* **(I)**, or *lipl-1* **(J)** RNAi bacteria, prior to OP50 or PA14 exposure. All experiments were repeated at least three times. Column data with plots are presented as mean ± SEM. Statistical significance was determined by log-rank test for survival assay or Kruskal–Wallis with Dunn’s multiple comparison test for **(I, J)**. *P < 0.05; **P < 0.01; ***P < 0.001; n.s., not significant.

To test if lysosomal signaling is involved in INX-14/GLP-1-mediated gut defense against pathogen infection, we assayed the survival rate of animals upon RNAi knockdown of the four lysosomal genes, *hlh-30, lipl-1, lipl-2*, and *lipl-3*. We found that animals treated with each RNAi showed an increased sensitivity to PA14 infection (**Figures S4A–S4D**). To further investigate in which tissues the lysosome functions to defend against PA14 infection, we performed survival assay using tissue-specific RNAi. We found that *hlh-30* RNAi (**Figures S4E–S4H**), or *lipl-1* RNAi (**Figures S4I–S4L**) in the intestine (VP303) (**Figures 6A, B**), but not in the neuron (TU3311) (**Figurea S4E**, **S4I**), muscle (WM118) (**Figures S4F, S4J**), germline (DCL569) (**Figures S4G , S4K**) or hypodermis (NR222) (**Figures S4H, S4L**), exhibited enhanced sensitivity to PA14 infection, suggesting that a lysosomal pathway acts in the intestine for PA14 defense. To examine the genetic relationship between gonadal INX-14/GLP-1 signaling and the lysosomal pathway, we examined the survival rate of wild-type animals or *inx-14(ag17)* mutants fed with control, *hlh-30* or *lipl-1* RNAi. We found that the enhanced susceptibility of *hlh-30* (**Figures 6C, E**) or *lipl-1* (**Figures 6D, F**) RNAi-treated animals was converted to enhance resistance to PA14 infection. This effect was indistinguishable from the enhanced resistance of *inx-14(ag17)* (**Figures 6C, D**) or *glp-1(e2141)* (**Figures 6E, F**) mutants upon PA14 infection. Consistently, knocking down *inx-14* or *glp-1* by RNAi in the *hlh-30(tm1978)* (**Figures S5A**, **S5B**) or *lipl-1(tm1987)* (**Figures S5C**, **S5D**) mutants completely suppressed the enhanced susceptibility of *hlh-30(tm1978)* (**Figures S5A–S5B**) or *lipl-1(tm1987)* (**Figures S5C–S5D**) single mutants. Moreover, we did not observe any alteration of *inx-14* and *glp-1* mRNA levels following *hlh-30* or *lipl-1* RNAi (**Figures S5E**, **S5F**). Collectively, these data suggested that one or more biological processes may act in parallel to a lysosomal pathway downstream of the INX-14/GLP-1 signaling axis to regulate gut defense.

To understand whether lysosome-mediated gut defense requires PMK-1/p38 signaling, we examined the effects of *hlh-30* or *lipl-1* RNAi treatment on the survival of PMK-1/p38 pathway-dependent gene mutants. These experiments revealed that the enhanced sensitivity of *hlh-30* (**Figures 6G**, **S6A**, **S6B**) or *lipl-1* (**Figures 6H**, **S6C**, **S6D**) RNAi-treated animals to PA14 infection was further enhanced by *nsy-1* (**Figures S6A**, **S6C**), *sek-1* (**Figures S6B**, **S6D**), or *pmk-1* mutation (**Figures 6G, H**). Additionally, RT-qPCR analyses showed that the expression of six PMK-1/p38-dependent genes was increased in the *hlh-30* or *lipl-1* RNAi-treated animals, or upon PA14 infection (**Figures 6I, J**). To address if *pmk-1* affects the lysosome pathway, we investigated the nuclear translocation of HLH-30::GFP in *pmk-1* RNAi-treated animals and observed that the proportions of nuclear localization of HLH-30 between control and *pmk-1* RNAi-treated animals were comparable, while the proportion of HLH-30::GFP nuclear localization dramatically increased upon PA14 infection (**Figures S6E**, **S6F**). Consistently, the mRNA levels of *hlh-30* and *lipl-1* in a *pmk-1(km25)* mutant were comparable to that of wild-type animals (**Figure S6G**). Together, these data suggested that the lysosomal pathway acts in the intestine and downstream of PMK-1/p38 to defend the intestine against pathogen infection (**Figure S6H**).

## Discussion

### The reproductive tract suppresses gut defense via gap junction

Gap junctions, essential players for cell-cell communication, are made of two hemichannels (commonly called connexons), present on adjacent cell. Found in almost all cells, gap junctions play a pivotal role in many physiological and cellular processes, and have even been linked to the progression of diseases, such as arrhythmia, neurosensory deafness, cancer (66–68). Gap junctions play important roles in antibody production, specific immune responses, sensing danger in tissue and transferring electrical, metabolic and immunological information by the immune system (69). However, their roles in intestinal innate immunity remain unclear. Here, we systematically investigated the role of gap junction genes that affect *C. elegans* sensitivity to *Pseudomonas aeruginosa* PA14 infection. We demonstrate that *inx-14*, a heterotypic and heteromeric gap junction subunit, suppresses intestinal defense, as mutants lacking this gene showed strongly increased resistance to PA14 infection. Recent studies have demonstrated that INX-14 is widely expressed in the germline, neurons and muscle (34, 40). To determine the tissue in which INX-14 functions, we performed tissue-specific RNAi and rescue assays and remarkably found that INX-14 primarily acts in the germline. A recent study suggested that one class of heterotypic and heteromeric gap junctions (composed of INX-8 and INX-9 in somatic cells and INX-14 and INX-21 in the germline) is required for the proliferation and differentiation of germline stem cells (16), while another class (composed of somatic INX-8 and INX-9 and germline INX-14 and INX-22) is required for the negative regulation of oocyte meiotic maturation (16).

Previous work demonstrates that insulin/IGF-1 signals from the reproductive system shorten longevity. In *C. elegans*, prevention of germline stem cell proliferation results in the extension of lifespan, which requires the activity of DAF-16, a member of the forkhead/winged-helix family of transcriptional regulators (70, 71). Here we showed an example in which the reproductive system’s gap junction suppresses the intestine against pathogen infection. We have yet to determine if the other subunits INX-8, INX-9, INX-21, and INX-22 of the heterotypic and heteromeric gap junctions act together for intestinal defense.

In humans, the reproductive tissues secrete a variety of hormones such as estrogens and testosterone, which have profound effects not only on development and behavior, but also on immune and inflammatory responses in a cell autonomous way (2, 72). The reproductive system of *C. elegans* is one of the most sexually dimorphic tissues in the animal, with many components differing between hermaphrodites and males. Multiple studies demonstrated that germline removal in *C. elegans* or *Pristionchus pacificus* promotes longevity and increases innate immunity by activating the transcription factor DAF-16/FOXO that is repressed by insulin/insulin-like growth factor signaling (IIS) (53, 73–75). Here, we uncovered that an unexpected signaling axis involving INX-14/Gap junction and GLP-1/Notch in the gonad regulates intestinal defense in a cell non-autonomously manner.

### The Notch pathway transmits defense signaling from the reproductive tract to the intestine

To provide insight into the mechanisms underlying the regulation of intestine defense by gap junctions from the reproductive tract, we performed comprehensive transcriptome profiling and found that GLP-1/Notch specifically acts in the germline and downstream of INX-14 to suppress gut defense. Previous studies revealed that loss of heterotypic and heteromeric gap junctions INX-8/9/14/21 prevents germline development and gametogenesis (16, 48, 76). Remarkably, previous genetic epistasis experiments determined a role for the INX-8/INX-9:INX-14/INX-21 gap junction channels in germline proliferation independent of the GLP-1/Notch pathway (16), while we found that INX-14 acts in the same genetic pathway as GLP-1 for intestinal defense. In addition, DAF-7/TGF-β signaling acts in the germline stem cell niche to regulate the balance of proliferation versus differentiation independently of insulin/IGF-like receptor signaling (48). Here we found that DAF-7/TGF-β signaling is not required for INX-14-mediated intestinal defense. Finally, INX-14/GLP-1 signaling-dependent intestine defense is dependent on PMK-1/p38 but not insulin/IGF-like receptor signaling. Therefore, the effect of INX-14/GLP-1 signaling on intestinal defense is unlikely to be a secondary consequence of reproductive development defects.

Gap junctions are intercellular communication channels that allow a direct transfer of signaling molecules from cell to cell (77). In vertebrate, various communication mediators such as dsDNA, Ca^2+^, cAMP, and IP3 by gap junctions have been implicated in the activation of innate immunity (78–80). It’s quite interesting to investigate how the gap junction INX-14 transmit defense signaling from gonad to intestine.

Activation of Notch signaling relies on the ability of a ligand to trigger receptor proteolysis resulting in the release of an active Notch fragment. The intramembrane proteolysis is involved in receptor activation. After its release by proteolysis from a membrane tether, the Notch intracellular domain (NICD) translocates to the nucleus and associates with a DNA binding protein to activate expression of downstream target genes. This core signal transduction known as the “canonical” pathway is used in most Notch-dependent processes (47). *C. elegans* has two Notch receptor genes, *lin-12* and *glp-1*, and they play important roles in cell fate decisions during development, dauer formation and maintenance, and oocyte growth and cell fate reprogramming. The release of the intracellular domain requires two proteolytic cleavage steps. The first cleavage is triggered by binding of a ligand of the DSL family and exposing a cleavage site (“site 2”, S2) in the ectodomain accessible to ADAM (A Disintegrin And Metalloprotease domain, ADAM) family proteases SUP-17/ADAM10 and ADM-4/TACE (TNFβ-converting enzyme, TACE), which function redundantly as S2 proteases. The second cleavage is a substrate for γ-secretase, which cleaves within the transmembrane domain at “site 3”. The intracellular domains released from its transmembrane tether translocate to the nucleus. In the nucleus, the intracellular domain forms a complex with a sequence-specific DNA binding protein of the CSL family and a co-activator of the Mastermind family to promote target gene expression (81). In our case, loss-of-function of either Notch ligand gene *apx-1* or *lag-2*, or together (**Figures S3C–S3E**), or knocking down S2 protease genes *sup-17* and *adm-4* by RNAi failed to phenocopy the survival rate of Notch receptor *glp-1* mutants upon PA14 infection. It will be quite interesting to investigate the mechanisms underlying how Notch signaling transmits defense signaling to the intestine in future.

### A lysosomal pathway is involved in intestinal defense

Lysosomes are highly dynamic organelles that are responsible for macromolecule degradation and catabolite recycling (82). They participate in many cellular processes including metabolic signaling, gene regulation, immunity, plasma membrane repair and cell adhesion and migration. Lysosomal dysfunction is implicated in the pathogenesis of diseases such as neurodegeneration, metabolic disorders, and cancer (63). The mammalian transcription factor EB (TFEB) is known to control the transcription of autophagy and lysosomal biogenesis genes in response to nutritional stress (83). Previous studies demonstrated that *C. elegans* HLH-30/bHLH, a basic helix-loop-helix transcription factor orthologous to mammalian TFEB, plays an important role in longevity (84) and host response to infection (65).

Our RNA-Seq data revealed that lysosome pathway genes were enriched and upregulated by germline-specific loss of *inx-14*. We further found that nuclear accumulation of HLH-30 was robustly induced by *inx-14*, or *glp-1* RNAi under OP50 feeding or PA14 infection conditions, as compared to wild-type fed on OP50. These results suggest that the Gap junction/Notch signaling axis suppresses a lysosomal pathway via an unidentified intercellular signaling between gonad and intestine. It is also possible that Gap junction/INX-14 regulates calcium signaling, malonyl-CoA (85, 86) or other messenger such as cyclic GMP (24) that triggers to cleave and activate Notch/GLP-1 (47) in the gonad, which consequently transduces activated Notch/GLP-1 signal to epithelial cells of the intestine and trigger gut lysosome pathway. The activated lysosome may stimulate PMK-1 activation and promote lysozymes for pathogen defense in the intestine. Our survival data showed that knocking down *hlh-30* or *lipl-1* by RNAi caused weak enhanced susceptibility to PA14 infection, while either *inx-14* or *glp-1* mutants exhibited strong enhanced resistance. However, the susceptibility caused by RNAi knockdown of *hlh-30* or *lipl-1* in *inx-14(ag17)* or *glp-1(e2141)* mutants are indistinguishable from that of *inx-14(ag17)* or *glp-1(e2141)* single mutants. These data suggest that *inx-14* and *glp-1* are epistatic to *hlh-30* and *lipl-1*. Since gonadal INX-14 controls many other biological processes, including peroxisome, longevity regulating pathways, or carbon metabolism, these pathways may function redundantly or in parallel to each other, and downstream of the INX-14/GLP-1 signaling axis for the intestinal defense. Hence, if one of these signaling pathways, for instance the lysosome pathway, is removed, we might not observe an obvious effect on the survival of animals upon PA14 infection, which is consistent with our findings in this study.

## Materials and methods

### C. elegans strains

Strains were maintained at 20˚C on nematode growth medium (NGM) plate seeded with *Escherichia coli* OP50 as a food source using standard procedures, except *glp-1(e2141)* mutants that were raised at 15˚C (87). The N2 Bristol strain of *C. elegans* was used as the wild-type reference strain. The following mutant alleles and transgenes were used in this study: *inx-8(gk42), inx-9(gk293), inx-14(ag17), inx-21(ok2524), inx-22(tm1661), inx-19(ky634), unc-7(e5), unc-9(e101), eat-5(ad1402), che-7(ok2373), inx-6(rr5), inx-2(ok376), inx-5(ok1053),inx-7(ok2319), inx-10(ok2714), inx-11(ok2783), inx-16(ox144), inx-18(ok2454), inx-20(ok681), inx-1(tm3524), inx-15(tm3394), inx-17(tm3292), inx-14(ag17);pmk-1(km25), glp-1(e2141), DCL569 mkcSi13 [sun-1p::rde-1::sun-13’UTR+unc-119(+)];rde-1(mkc36), VP303 rde-1(ne219);kbIs7 [nhx-2p::rde-1+rol-6(su1006)], WM118 rde-1(ne300);neIs9 [myo-3::HA::RDE-1 + rol-6(su1006)], TU3311 uIs60 [unc-119p::YFP + unc-119p::sid-1] pmk-1(km25), nsy-1(ag3), sek-1(km4), glp-1(e2141), hlh-30(tm1978), lipl-1(tm1987)*, AY101 *acIs101 [F35E12.5p::GFP + rol-6(su1006)]*, SAL144 *pha-1(e2123);denEx22 [K08D8.5::GFP + pha-1(+)]*, *unc-17(e245), unc-25(e156), unc-13(e51)*, MAH235 *sqIs19 [hlh-30p::hlh-30::GFP + rol-6(su1006)], unc-17(e245), unc-13(e51), unc-25(e156), qxIs520[Pvha-7::LAAT-1::GFP].*

Transgenic strains generated for this study to express INX-14 under the control of endogenous or tissue-specific promoters in an *inx-14(ag17)* genetic background are: *inx-14(ag17);aijEx186[pXZ20;pCFJ90]* and *inx-14(ag17);aijEx219[pXZ23; pCFJ90].*

The bacterial strains used in this study were *Escherichia coli* OP50, *E. coli* HT115, and *Pseudomonas aeruginosa* PA14 (6). All the bacteria were grown in Luria-Bertani (LB) broth at 37°C and seeded on nematode growth medium (NGM) plates as the food source unless otherwise indicated.

### Germline transformation

Transgenic animals were created by microinjection of plasmid DNA into the gonad of young adult *C. elegans* animals as described previously (88). For specific rescue transgenes of *inx-14(ag17)*, the plasmid DNA of pXZ20 (*Ppgl-1::inx-14::SL2::GFP::unc-54 3’ UTR*), or pXZ23 (*Pges-1::inx-14::SL2::GFP::unc-54 3’ UTR*) was injected at 20 ng μl^-1^ with co-injection marker at 5 ng μl^-1^, and 1 kb ladder up to 100 ng μl^-1^.

### Plasmid constructions

The plasmids were constructed by using isothermal assembly (89), unless stated otherwise. The following plasmids were created in this study:

pXZ20: *Ppgl-1::inx-14::SL2::GFP::unc-54 3’ UTR*. To assemble pXZ20, a 207 bp fragment of the germline-specific promoter *Ppgl-1* and a 1.6 kb genomic DNA fragment of the *inx-14* coding region (from initiation codon ATG to stop codon TAA) were amplified from N2 genomic DNA and fused to an SL2::GFP sequence and *unc-54* 3’UTR (from pNP403). This assembly was inserted into pJET1.2 (Clone JET PCR Cloning kit, Thermo Scientific, K1232#) using isothermal assembly (89).

pXZ23: *Pges-1::inx-14::SL2::GFP::unc-54 3’ UTR*. To assemble pXZ23, a 2.5 kb fragments of intestine-specific promoter *Pges-1* and a 1.6 kb genomic DNA fragment of the *inx-14* coding region (from initiation codon ATG to stop codon TAA) were amplified from N2 genomic DNA and fused to an SL2::GFP sequence and *unc-54* 3’UTR (from pNP403). This assembly was inserted into pJET1.2 (Clone JET PCR Cloning kit, Thermo Scientific, K1232#) using isothermal assembly (89).

### Pathogen PA14 killing assay

The killing assay by *P. aeruginosa* PA14 infection was performed as described previously (6) with minor modifications. The overnight culture of *P. aeruginosa* PA14 was seeded onto modified 35 mm-diameter NGM plates (0.35% instead of 0.25% peptone) to cover the entire surface of NGM agar (6). These slow-killing assay plates were dried at room temperature and incubated at 37°C for 24 h and equilibrated at 25°C for 8-24 h before being used for the assay. Synchronized L4 worms were transferred to the *P.aeruginosa* PA14 plates and cultivated at 25°C. Worms were counted for death events every 12 hours for survival and transferred to fresh slow killing plates daily. Animals were considered dead if they failed to respond when the head or the tail was touched using an eyebrow. 5-Fluoro-2’-deoxyuridine (FUdR, 50 mg/ml) (Sigma, F0503) was added to plates to prevent the growth of progeny and “bagging”. Animals that crawled off the plate or died from vulva bursting were censored. All survival assays were performed in at least three independent replicates.

### RNA interference in *C. elegans*

RNA interference (RNAi) was performed by feeding worms with *E. coli* strain HT115 (DE3) expressing double-strand RNA homologous to a target gene as previously described (90). RNAi clones were obtained from the Ahringer RNAi library. Bacteria were grown at 37˚C overnight in LB liquid medium containing 100 g/ml ampicillin, then 1 mM isopropyl β-D-1-thiogalactopyranoside (IPTG) was added to induce dsRNA production for 3-5 hours. 300 µl *E. coli* HT115 bacteria carrying either the desired RNAi or the empty vector pL4440 were then seeded onto NGM with 100 g/ml ampicillin, hood-dried in a dark environment and cultured overnight at 25 ˚C. Adult animals were then transferred onto RNAi bacterial lawns to lay eggs at 20°C for 2 hours and removed after laying eggs. The eggs hatched were allowed to develop at 20°C to reach the L4 stage for subsequent experimental use.

### Tissue specific RNAi

Tissue-specific gene knockdown was performed as described previously (91, 92) with minor modifications using the strains targeting RNAi to the germline (DCL569), intestine (VP303), neurons (TU3311), muscle (WM118) or epidermis (NR222). Synchronized eggs of DCL569, VP303, TU3311, WM118, and NR222 were cultured on NGM plates seeded with *E. coli* HT115 RNAi clones at 20°C until the progeny reached the L4 stage. These animals were then transferred to NGM plates with full-lawn PA14 at 25°C for survival assays.

### RNA preparation and quantitative real-time PCR

Synchronized larval L4 worms were transferred onto NGM plates seeded with OP50 or PA14 maintained at 25°C for 24 hours. Animals were washed off and collected, quickly rinsed once with M9 solution, and then re-suspended in TRIzol (Transgen, ER501-01-01) for total RNA extraction or frozen by liquid nitrogen and then stored at -80°C for subsequent experiments. Total RNA was obtained using TransZol Up Plus RNA Kit (Transgen, ER501-01) and cDNAs were synthesized using the TransScript All-in-One First-Strand cDNA Synthesis SuperMix (Transgen, AT341-02). cDNA aliquots were stored at -80°C for subsequent experiments. qRT-PCR was performed using SYBR PCR Master Mix (Transgen, AQ141-02) on a Bio-Rad CFX96 real-time PCR machine. Relative fold-changes for transcripts were calculated using the comparative CT (2^-ΔΔCT^) method and normalized to *tba-1*/tubulin. All experiments were performed at least three times independently. Primers used for these experiments are listed in Supporting **Table S3**.

### RNA-Seq experiment

Total RNA (1 µg) was obtained as described above from the germline-specific DCL569 RNAi strain fed with L4440 empty vector control, *inx-14* RNAi (prior to PA14 infection), or OP50 for 24 hours. These samples was used as input material for RNA sample preparation. Sequencing libraries were generated using the NEBNext® Ultra™ RNA Library Prep Kit for Illumina® (NEB) following manufacturer recommendations, and index codes were added to attribute sequences to each sample. Library preparations were sequenced on an Illumina NovaSeq 6000 platform using 150 bp paired-end reads.

Clean reads were mapped to the worm reference genome (Wbcel235) using HISAT2 (93). Uniquely mapped reads were summarized to WormBase annotated genes using feature Counts (94). The raw gene expression matrix was imported into the R environment for further processing and analysis. Genes with low read counts (less than 50 reads in all eight libraries) were filtered out, leaving sets of ~15,000 genes to test for expression differences between conditions. Different expression analyses were carried out using R package DESeq2 (95). Differentially-expressed genes (DEG) were identified based on two criteria: FDR (False discovery rate using Benjamini-Hochberg adjusted p-values) <0.01 and absolute value of log2(Fold Change) >1. Hierarchical clustering was performed for all leaving sets of genes using Pearson correlations. Differentially-expressed genes were used to perform KEGG pathway enrichment analyses using R package cluster Profiler (96). The top 20 terms with the lowest adjusted p-values were considered here.

### Defecation assay

The defecation cycle length was measured on synchronized young adult hermaphrodites on standard NGM plates at 20°C. Animals were transferred to fresh plates and were allowed to adapt for at least 10 min before observation. Measurements were obtained from animals with OP50 feeding and the duration of the cycle was defined as the time from one expulsion to the next. Ten defecation cycles were measured for each individual. To determine the percentage of successful enteric muscle contractions per defecation cycle, we scored for the occurence of an enteric muscle contraction following a posterior body contraction (97).

### Brood size

Brood size was measured by picking single L4 worm to a 3.5 cm NGM plate, maintained at 20°C. Each worm was transferred to a new NGM plate every day and the previous plate was kept at 20°C for two more days until the number of live progeny was scored. This procedure was repeated until no eggs were laid anymore on NGM plate.

### Pharyngeal pumping behavior assay

Pharyngeal pumping was assayed by recording a 1 minute movie focusing on the pharynx of young adult worms using the DIC optics of Multi-Purpose Zoom Microscope (AZ100, Nikon). One opening and one closing of the terminal bulb (TB) was regarded as a complete pumping sequence. Pharyngeal pumping rates were reported as number of pumps per minute.

### Quantification of intestinal PA14 CFU

The quantification procedure of intestinal PA14 colony forming units (CFU) was modified from a previous study (39). Briefly, after exposure to *P. aeruginosa* PA14 for 24 hours, ten worms were transferred onto a fresh 3.5 cm NGM plate with M9 solution containing 25 mM sodium azide (Amresco, 0639) to paralyze the worms. Worms were then washed once by M9 solution containing 1 mg/ml ampicillin and 1 mg/ml gentamicin (Solarbio, G8170, China) and incubated for 0.5 h to kill bacteria attached to the surface of the animals on the plate. After worms were lysed with a motorized pestle, serial dilutions of the lysates (10^-1^, 10^-2^, 10^-3^, 10^-4^) were plated onto Luria-Bertani plates containing 100 g ml^-1^ rifampicin (Solarbio, R8011) and incubated overnight at 37°C to select for PA14 colony. PA14 colonies were counted to determine CFU per worm. At least three independent replicates were performed for each condition.

### Imaging and fluorescence quantification

To quantitatively analyze the fluorescence intensity of PMK-1/p38-dependent gene reporters, *F35E12.5p::GFP* or *K08D8.5p::GFP* images were acquired using a digital automated microscope (Nikon, AZ100, Japan). Synchronized eggs were grown to the L4 stage by feeding on L4440 vector control or gene-specific RNAi bacteria, prior to *E. coli* OP50 feeding or *P. aeruginosa* PA14 infection for 12 to 24 h. Animals were paralyzed with 1 mM sodium azide (Sigma) in M9 buffer and mounted on 2% agarose pads. To evaluate the fluorescence level in different genetic backgrounds, each genotype was analyzed on three different days, and the data were pooled. Data are presented as a percentage of the mean fluorescence relative to that of the wild-type or vector control. All results of fluorescence quantification are presented as means and SEM.

### Visualization of bacterial accumulation assay

Overnight culture of *P. aeruginosa* PA14 expressing GFP (PA14::GFP) were seeded on NGM plates and incubated at 37°C for 24 h. Synchronized L4 larvae were transferred to these plates and cultured at 25°C for 24 h. Before the examination of bacterial accumulation in the intestine and the pharyngeal lumen of animals, worms were washed in M9 buffer to eliminate fluorescent bacteria attached to the cuticle of worms. The visualized fluorescence of PA14::GFP was observed by using ultra-high resolution spectral confocal microscope (Olympus, fv1000, Japan), and the fluorescence intensity was quantified using Image-J software (v1.8.0, NIH).

### Green fluorescent bead intake assay

This assay was done by feeding synchronized worms with 0.5 μm green fluorescent polystyrene beads (44). Overnight *P. aeruginosa* PA14 cultures were mixed with the beads at a ratio of 25:1 and 100μl of mixture were placed onto the plate and incubated at 37°C overnight. Young adult animals were transferred to the plate and incubated at 25°C for 2 hours. Worms were then washed in M9 buffer with 25 mM sodium azide to remove the fluorescent beads that adhere to the body surface, and mounted on glass slides for imaging using the a multi-purpose zoom microscope (AZ100, Nikon). The fluorescence intensity of the area containing the GFP fluorescent beads was analyzed using Image-J software (v1.8.0, NIH).

### Avoidance assay

The PA14 avoidance assay was performed with minor modification as previously described (39). A 20 μL drop of *P. aeruginosa* PA14 culture was seeded in the center of 6.0 cm NGM plates and cultured at 37°C for 24 h. Thirty L4 larva were placed in the center of each PA14 lawn on the NGM plate. The number of worms within the PA14 lawn was counted at 2, 4, 6, 8 and 10 h post-exposure.

### Lifespan assay

Synchronized L4 stage worms were transferred to the OP50 lawn of NGM plate. Animals were scored for survival daily and transferred to fresh plates every 2-3 days. Animals were considered dead if they failed to respond to the repeated touch of a platinum wire. Those that died of vulva burst or crawling off plates were censored. The assay was performed at 25°C.

### Western blotting

Worm lysates were prepared using lysis buffer in the presence of protease inhibitors (Sigma-Aldrich, USA) or protease and phosphatase inhibitors (Sigma-Aldrich, USA). Proteins were boiled for 10 min at 98°C before gel electrophoresis. For the western blotting, we used primary anti-phospho-p38 MAPK rabbit polyclonal antibody (Cell Signaling Technology, 4511, USA) at a 1:1,000 dilution, or anti-β-actin mouse monoclonal antibody (Transgen, HC201-01, China) at a 1:3,000 dilution. A horseradish peroxidase (HRP)-conjugated goat anti-rabbit (Transgen, HS101-01, China), or anti-mouse (Transgen, HS201-01, China) were used as secondary antibody at a 1:2,000 or 1:5,000 dilution, respectively. Images of western blot membranes were captured using an imaging system (MicroChemi 4.2, DNR Bio Imaging Systems). All western blotting experiments were repeated at least three times independently.

### Magic red staining

Magic Red stain (Immuno Chemistry Technologies #938, Bloomington, Minnesota) is cleaved by Cathepsin B and generates a red fluorescent substrate in functional lysosomes (59). Magic Red stain was prepared in 260x DMSO stock following the manufacturer’s instructions. This 260x stock was diluted with 60 μl M9 buffer, spread onto a well of a 24-well-plate containing 1 ml NGM and dried at room temperature. Synchronized eggs were grown to the L4 stage by feeding on L4440 vector control or gene-specific RNAi bacteria, prior to *E. coli* OP50 feeding or *P. aeruginosa* PA14 infection for 12 to 24 h. L4 worms were transferred onto Magic Red dye-containing NGM plate seeded with OP50 or PA14 and cultured overnight at 25 ˚C. Worms were photographed with ultra-high resolution spectral confocal microscope (Olympus, fv1000, Japan) and quantified by Image-J.

### Statistical analysis

Data were analyzed with Prism 8.0 (v8.4.3, GraphPad Software, Inc.) and SPSS® Statistics Version 25 (IBM, USA). For statistical analyses of all killing and life span assays, Log-rank (Kaplan-Meier) method was used to calculate p-values. The exact p-values for all survival assay are listed in Supporting **Table S1**. Column data with plots are presented as mean ± SEM. Statistical significance was determined by log-rank test for survival assays, or unpaired Student’s t-test for comparison between column data. The exact p value for all statistical analysis of quantitative RT-PCR are listed in Supporting **Table S2**.

## Data availability statement

The datasets presented in this study can be found in online repositories. The names of the repository/repositories and accession number(s) can be found below: GSE216879 (GEO). Codes are available at 10.24433/CO.2273316.v1 (Code Ocean).

## Ethics statement

The manuscript presents research on animals that do not require ethical approval for their study.

## Author contributions

XZ and HT conceived ideas and designed experiments. XZ, YW, ZC, and ZW performed experiments and analyzed data. XZ and HT wrote the manuscript with input from all authors. All authors reviewed and edited the manuscript.
