# Supplementary material for: A gonadal gap junction INX-14/Notch GLP-1 signaling axis suppresses gut defense through an intestinal lysosome pathway
Source: Front Immunol. 2023 Oct 19;14:1249436. doi: 10.3389/fimmu.2023.1249436 (PMC10620905; doi:10.3389/fimmu.2023.1249436)
Supplement: Supplementary file 1 [file Presentation_1.pdf]

Supporting Information for

A Gonadal Gap Junction/Notch Signaling Axis Suppresses Gut Defense through an Intestinal Lysosome Pathway

Xiumei Zhang,[1,#] Yirong Wang,[1,#] Zixin Cai,[1,#] Zhiqing Wan,[1] Yilixiati Aihemaiti,[1] Haijun Tu[1,*]

[1] State Key Laboratory of Chemo/Biosensing and Chemometrics, College of Biology, Hunan University, 410082 Changsha, Hunan, China.

# These authors contributed equally to this work.

* Correspondence author: Haijun Tu

Email: haijuntu@hnu.edu.cn

Supporting Information includes:

Supplemental figures S1 to S6

Supplemental tables S1 to S3

Supplemental datasets S1 to S3

Supplemental figures

Figure S1

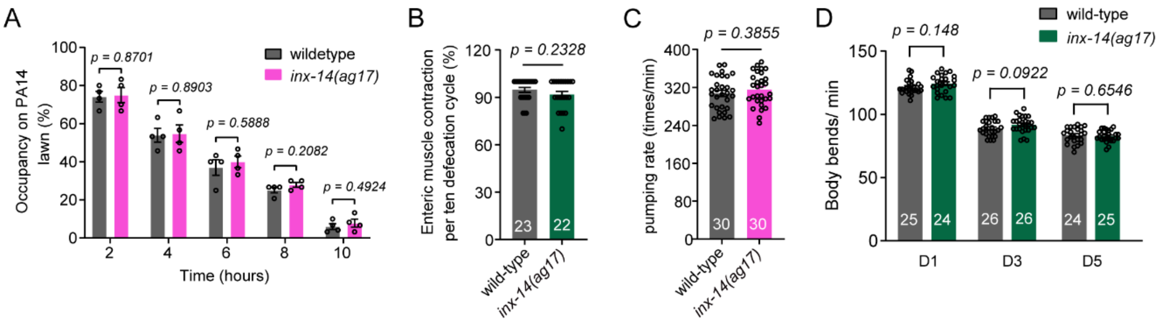

Fig. S1. *inx-14* mutation has no impact on *C. elegans* locomotor and feeding-related behaviors. (A) PA14 lawn occupancy of wild-type and *inx-14(ag17)* mutants assayed at five time points. (B) and (C) The percentage of enteric muscle contractions per ten defecation cycles (B) and pumping rates (C) of wild-type and *inx-14(ag17)* worms are indistinguishable. (D) Body bends of wild-type and *inx-14(ag17)* mutants at different adult stages (D1, D3, D5). All experiments were repeated at least three times. The number of animals analyzed is indicated and column data with plots are presented as mean ± SEM. Statistical significance was determined by unpaired Student's t test.

Figure S2

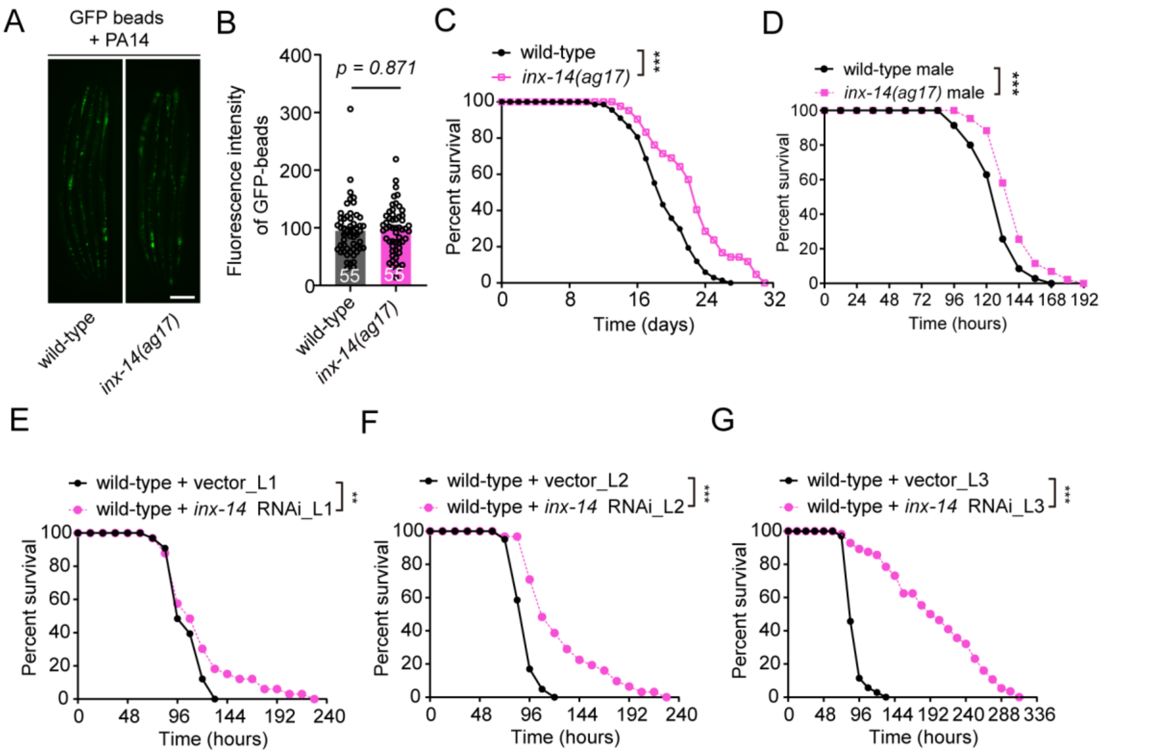

Fig. S2. Effects of *inx-14* inactivation on PA14 uptake, lifespan, and susceptibility of males and worm larvae to PA14 infection. (A) and (B) Representative images (A) and quantitative analysis (B) of GFP beads accumulated in the intestine of wild-type and *inx-14(ag17)* mutants. (C) Life span of wild-type and *inx-14(ag17)* mutants. (D) Survival of wild-type and *inx-14(ag17)* mutant males exposed to PA14. (E) – (G) Survival of wild-type animals at different larval stages L1 (E), L2 (F), and L3 (G) animals fed on vector control or *inx-14* RNAi bacteria, prior to PA14 infection. All experiments were repeated at least three times. The number of animals analyzed is indicated and column data with plots are presented as mean ± SEM in (B). Statistical significance was determined by log-rank test for survival assays, or unpaired Student's t test for (B). **P* < 0.01; ***P* < 0.05; ****P* < 0.001; n.s., not significant. Scale bar, 100 μm in (A).

Figure S3

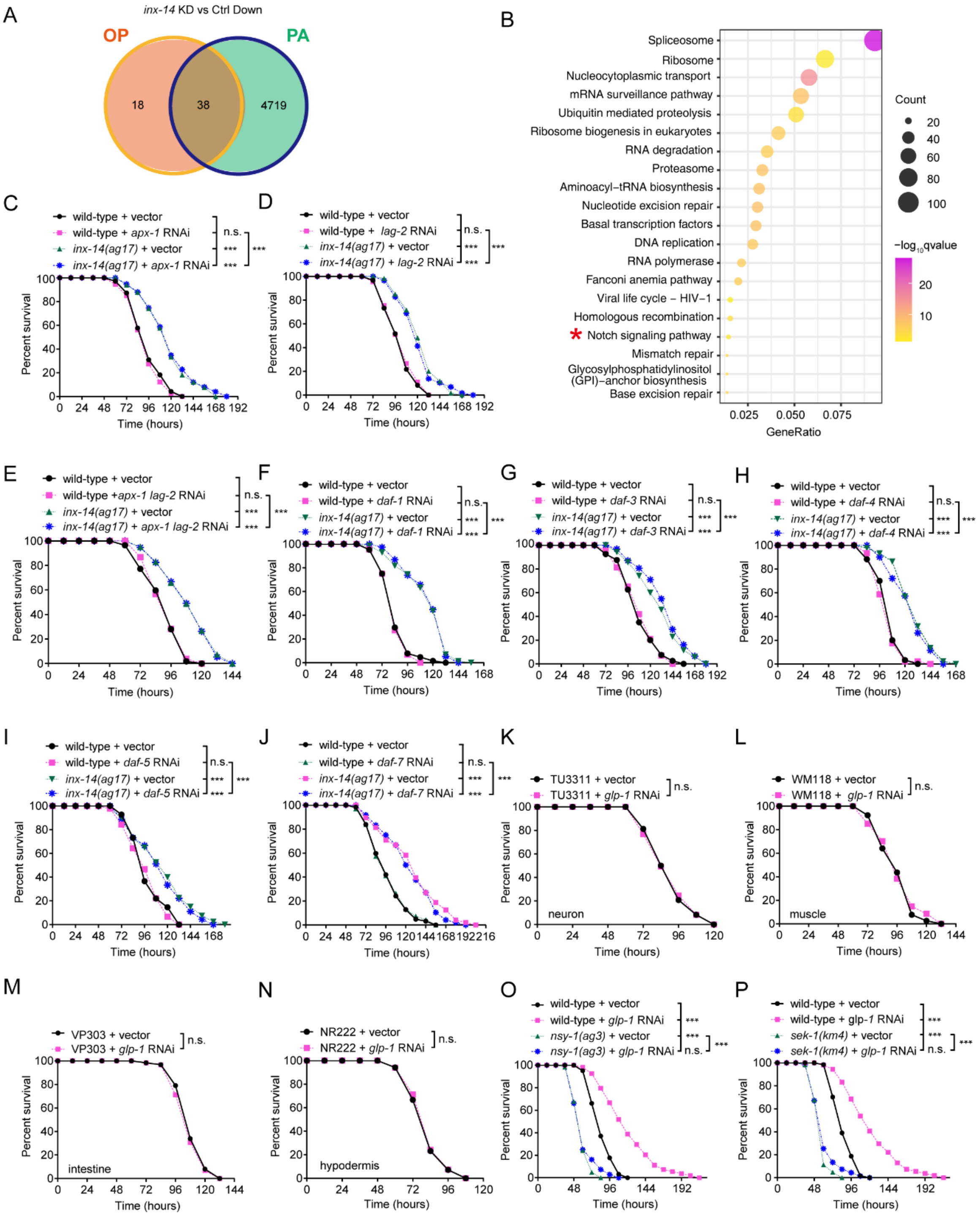

Fig. S3. GLP-1/Notch signaling but not TGF-β/DAF-7 acts downstream of INX-14 in the gonad to suppress intestinal defense. (A) Venn diagram of genes down-regulated upon germline-specific *inx-14* knock-down in OP50 or PA14 exposed animals. (B) KEGG pathway

enrichment analyses of the down-regulated genes in the wild-type animal with *inx-14* RNAi knockdown upon PA14 infection. The red asterisk marks the Notch signaling pathway. KEGG terms were sorted according to numbers of enriched DEG. (C-E) PA14 survival assays of wild-type and *inx-14(ag17)* mutants fed on vector control, or GLP-1/Notch component RNAi targeting *apx-1* (C) and *lag-2* (D) individually, or *apx-1 and lag-2* simultaneously (E). (F–J) Survival assays of wild-type and *inx-14(ag17)* mutant fed on vector control, or TGF-β/DAF-7 pathway RNAi targeting *daf-1* (F), *daf-3* (G), *daf-4* (H), *daf-5* (I), or *daf-7* (J), prior to PA14 exposure. (K–M) Survival assays upon tissue-specific RNAi knockdown of *glp-1* in neurons (TU3311) (K), muscle (WM118) (L), intestine (VP303) (M), or hypodermis (NR222) (N). (O) and (P) Survival of wild-type and the PMK-1/p38 pathway component genes *nsy-1* (O), or *sek-1* (P) mutants fed on vector control, or *glp-1* RNAi bacteria, respectively prior to PA14 exposure. All experiments were repeated at least three times. Statistical significance was determined by log-rank test for survival assays. ***$P < 0.001$; n.s., not significant.

Figure S4

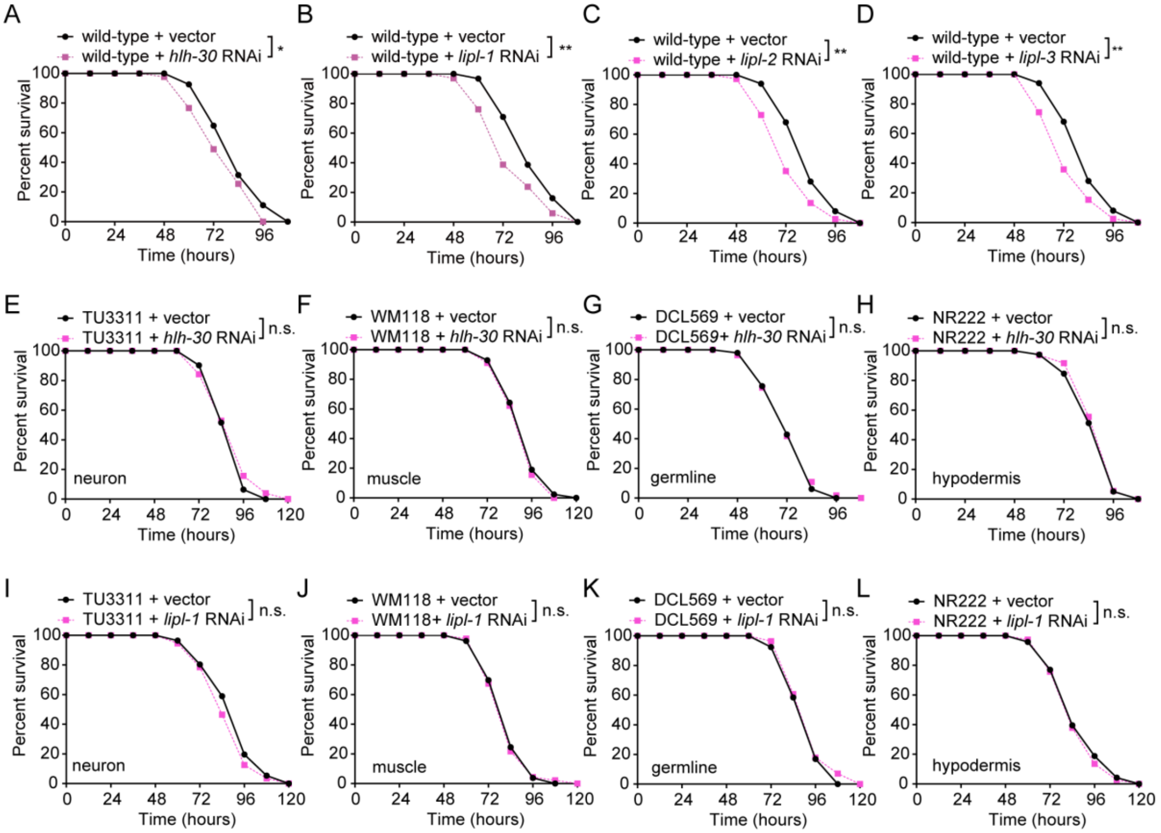

Fig. S4. Susceptibility to PA14 infection upon tissue-specific knock-down of lysosomal pathway genes *hlh-30* and *lipl-1* (A–D) PA14 survival assays of wild-type animals fed on vector control, *hlh-30* (A), *lipl-1* (B), *lipl-2* (C), or *lipl-3* (D) RNAi bacteria, prior to PA14 exposure. (E-H) Survival assays of tissue-specific RNAi knockdown of *hlh-30* in neurons (TU3311) (E), muscle (WM118) (F), germline (DCL569) (G), or hypodermis (NR222) (H). (I-L) Survival assays of tissue-specific RNAi knockdown of *lipl-1* in neurons (TU3311) (I), muscle (WM118) (J), germline (DCL569) (K), or hypodermis (NR222) (L). All experiments were repeated at least three times. Statistical significance was determined by log-rank test for survival assays. *$P < 0.01$; **$P < 0.05$; n.s., not significant.

Figure S5

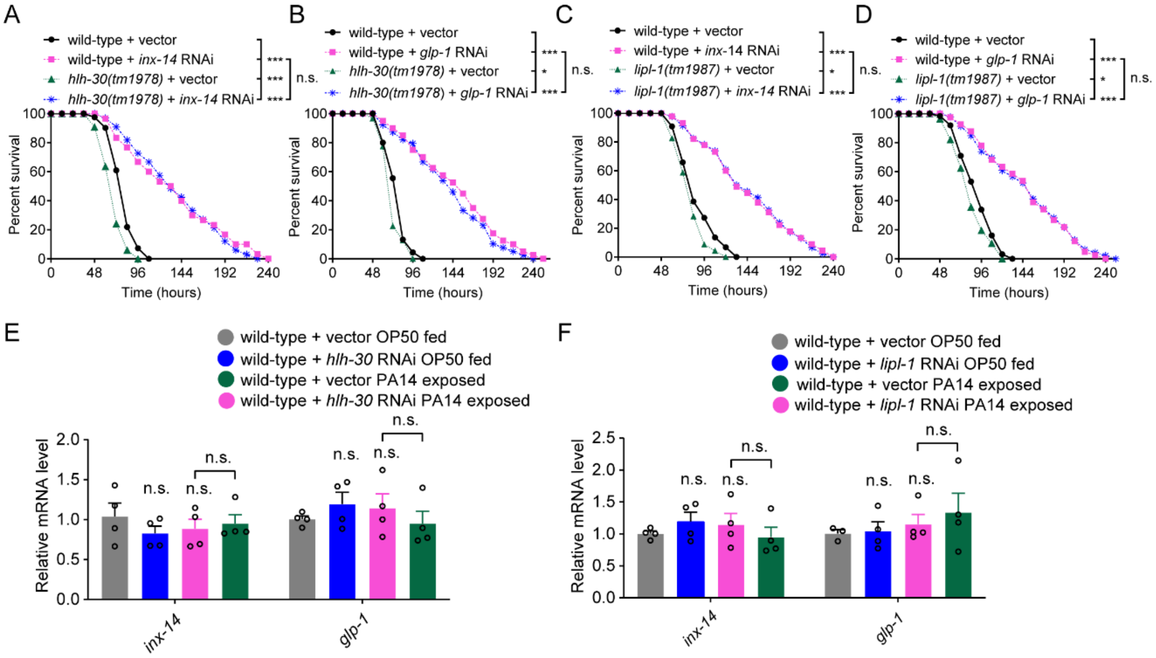

Fig. S5. The INX-14/GLP-1 signaling axis acts upstream of a lysosome pathway for intestinal defense. (A–D) Survival of wild-type and *hlh-30(tm1978)* (A and B) or *lipl-1(tm1987)* (C and D) mutants fed on vector control, *inx-14* (A and C), or *glp-1* (B and D) RNAi bacteria prior to PA14 exposure. (E and F) RT-qPCR analyses of *inx-14* and *glp-1* mRNA levels in wild-type animals fed on vector control, *hlh-30* (E), or *lipl-1* (F) RNAi bacteria, prior to OP50 or PA14 exposure.

Figure S6

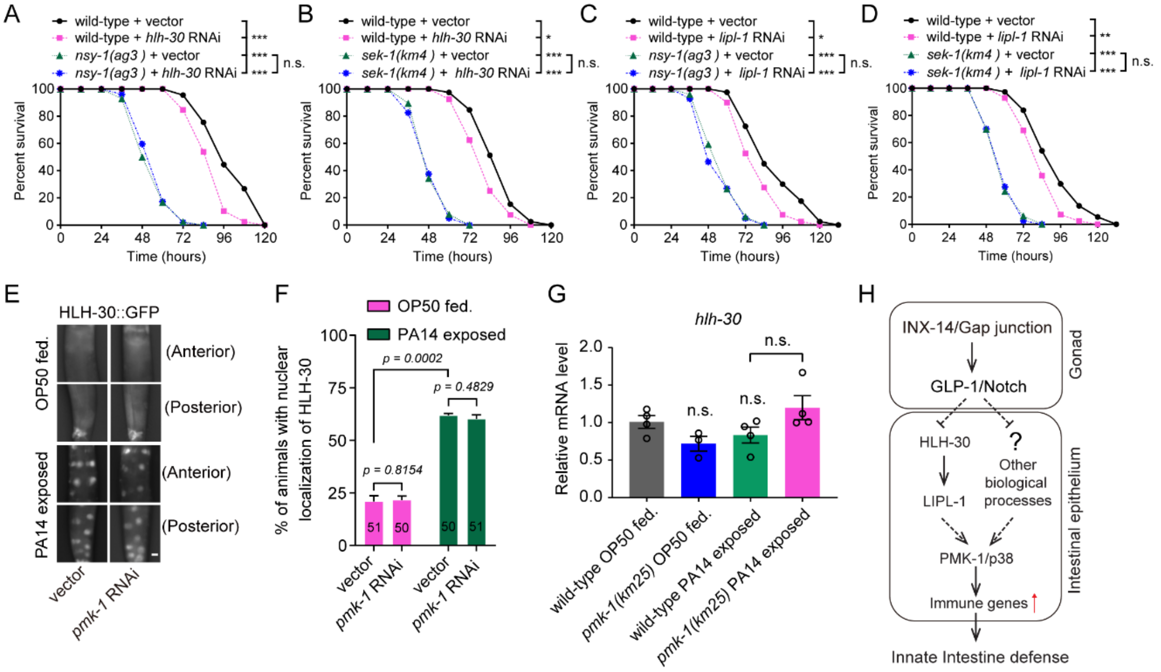

Fig. S6. HLH-30 and LIPL-1 act upstream of a PMK-1/p38 pathway for intestinal defense. (A-D) PA14 survival assays of wild-type and PMK-1/p38 pathway mutants *nsy-1(ag3)* (A and C), *sek-1(km4)* (B and D) fed on vector control, *hlh-30* (A and B), *or lipl-1* (C and D) RNAi bacteria, prior to PA14 exposure. (E) and (F) Representative images (E) and quantitative analyses (F) of nuclear accumulation of GFP-tagged HLH-30 (HLH-30::GFP) in wild-type animals fed on vector control and *pmk-1* RNAi bacteria prior to PA14 exposure. (G) RT-qPCR analyses of *hlh-30* mRNA levels in wild-type and *pmk-1(km25)* mutant animals fed on OP50 or exposed to PA14. (H) Schematic model illustrating a genetic pathway through which gonadal INX-14/GLP-1 signaling regulates innate intestinal defense through lysosome and PMK-1/p38 signaling. The putative site-of-action of each gene (gonad versus intestine) is denoted. All experiments were repeated at least three times. The number of animals analyzed is indicated in (F). Column data with plots are presented as mean ± SEM in (F) and (G). Statistical significance was determined by log-rank test for survival assays, or one-way ANOVA tests followed by Bonferroni's multiple comparison tests for (F) and (G). **P* < 0.01; ***P* < 0.05; ****P* < 0.001; n.s., not significant. Scale bar, 100 μm in (E).

Supplemental tables

Table S1. The exact *p* values of statistics for survival assay.

Table S2. The exact *p* values of statistics for quantitative real time PCR.

Table S3. The primers of the examined genes for quantitative real-time PCR.

Supplemental datasets (separate file)

Dataset S1. 4757 down-regulated genes in the germline specific RNAi knockdown strain DCL569 fed with *inx-14* RNAi bacteria versus vector control upon PA14 infection. The down-regulated genes were identified in the condition of padj < 0.05 and log2 Foldchange < -0.5 from RNAseq data.

Dataset S2. 2134 up-regulated genes in the germline-specific RNAi strain DCL569 fed on *inx-14* RNAi versus vector control bacteria upon PA14infection. The up-regulated genes were identified in the condition of padj < 0.01 and log2 fold change > 1 from RNAseq data.

Dataset S3. 48 genes enriched in lysosome pathway from KEGG.
